# Supplementary figures and images for: The association between Geographic Information System-based neighborhood built environmental factors and accelerometer-derived light-intensity physical activity across the lifespan: a cross-sectional study
Source: PeerJ. 2022 Apr 8;10:e13271. doi: 10.7717/peerj.13271 (PMC8997190; doi:10.7717/peerj.13271)

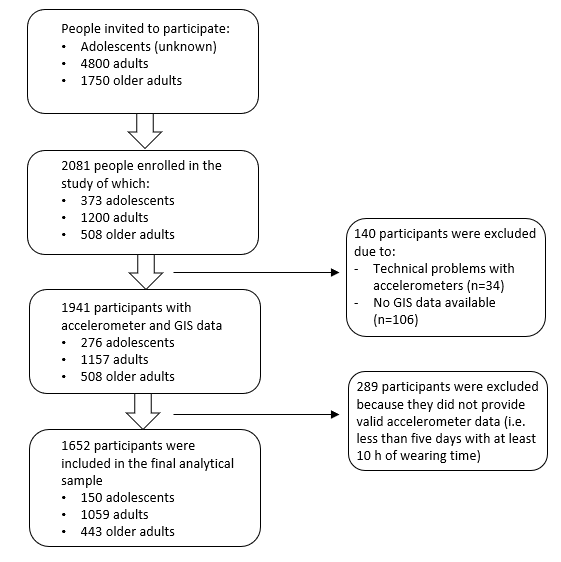

Supplement: File S1 [file peerj-10-13271-s003.docx]
